# A New Reliability Analysis Model of the Chegongzhuang Heat-Supplying Tunnel Structure Considering the Coupling of Pipeline Thrust and Thermal Effect

**DOI:** 10.3390/ma11020236

**Published:** 2018-02-03

**Authors:** Jiawen Zhang, Shaohui He, Dahai Wang, Yangpeng Liu, Wenbo Yao, Xiabing Liu

**Affiliations:** 1 School of Civil Engineering, Beijing Jiaotong University, Beijing 100044, China; jwzhangbjtu@163.com (J.Z.); 15115270@bjtu.edu.cn (D.W.); 14121142@bjtu.edu.cn (W.Y.); 10232025@bjtu.edu.cn (X.L.); 2 Beijing Teze Thermal Engineering Design Co. Ltd., Beijing 100027, China; ypliubjtu@126.com

**Keywords:** heat-supplying tunnel, large thrust, thermal effect, reliability, subset simulation method

## Abstract

Based on the operating Chegongzhuang heat-supplying tunnel in Beijing, the reliability of its lining structure under the action of large thrust and thermal effect is studied. According to the characteristics of a heat-supplying tunnel service, a three-dimensional numerical analysis model was established based on the mechanical tests on the in-situ specimens. The stress and strain of the tunnel structure were obtained before and after the operation. Compared with the field monitoring data, the rationality of the model was verified. After extracting the internal force of the lining structure, the improved method of subset simulation was proposed as the performance function to calculate the reliability of the main control section of the tunnel. In contrast to the traditional calculation method, the analytic relationship between the sample numbers in the subset simulation method and Monte Carlo method was given. The results indicate that the lining structure is greatly influenced by coupling in the range of six meters from the fixed brackets, especially the tunnel floor. The improved subset simulation method can greatly save computation time and improve computational efficiency under the premise of ensuring the accuracy of calculation. It is suitable for the reliability calculation of tunnel engineering, because “the lower the probability, the more efficient the calculation.”

## 1. Introduction

Heat-supplying tunnels are built for district heating pipelines in cold regions of the world, which is what distinguishes it from traffic and other municipal tunnels in its function and environment. High-temperature fluid transmission throughout the pipe leads to the temperature rising in heat-supplying tunnels. In addition, the pressure pipeline also produces a huge longitudinal thrust on the tunnel structure through the fixed brackets in the tunnel. The coupling of large thrust by the operating pipeline and thermal effect is always accompanied by cold and hot cycles and dry and wet alternation, which leads to the decrease of structural reliability and residual life. However, due to the fact that there is no direct contact between the public and the heat-supplying tunnel, the reliability and durability of these tunnels are seldom addressed by the public and scholars. In Beijing, for example, there were a large number of heat-supplying tunnels built in the 1990s. Heat-supplying tunnels in these regions were subject to structural degradation over time, causing latent risks to the above-grade traffic safety. Therefore, it is necessary to study the reliability of the tunnel lining structure under the coupling of large thrust by operating pipeline and thermal effect.

As far as reliability is concerned, the failure probability of the tunnel structure is often below 10^−4^ as a statically indeterminate system. A large number of sample points are needed in the calculation of small probability events, such as the traditional Monte Carlo method. The shortcoming of time-consuming and inefficient calculation usually limits its application in Tunnel Engineering. The subset simulation method can calculate the accurate reliability index by producing less sample points and was first proposed by Au in 2001 [1,2,3,4]. For the application and development of the subset simulation method, most scholars applied it to the reliability analysis in their field of research over the last ten years [5,6,7,8,9,10,11,12]. However, there were few application examples in tunnel engineering. This is because the general subset simulation method often needs to use the Monte Carlo sampling method to produce sample points. These sample points are actually the sample points of pseudorandom numbers (PRN) and there is a certain correlation between them. The convergence speed and accuracy of the reliability calculation could be affected by the PRN sample points. Taking the measures of improvement and making full use of the advantages of subset simulation to overcome its shortcomings, is a core focus of this paper, particularly in regard to applying it to the reliability calculation of heat-supplying tunnels.

To study the reliability of the heat-supplying tunnel in the operation period, the influence of the thermo-mechanical coupling environment on the lining structure during the operation period must be mastered. At present, the effect of thermo-mechanical coupling on tunnel structure is considered as follows: Ingason [13] conducted a 1:23 scale model to study the response of tunnel structure to fire under different longitudinal ventilation conditions; Megret [14] established a new semi-empirical model to obtain temperature field distribution under fire through theoretical derivation; Schrefler [15] and McGrattan [16] established a numerical tunnel fire model in a fluid-heat-stress field and then the evaluation method of concrete structure after fire was obtained; Xu Z.S. [17] obtained the mechanical changes of shield tunnels in fire induced by finite element analysis. In contrast with the above researches, although the temperature of the operating heat-supplying tunnel cannot reach a high temperature under fire, the pressurized pipeline laid inside it will cause huge longitudinal thrust to the tunnel structure through the fixed brackets. High-temperature environment leads to a long-term heat deterioration process for tunnel structures. This type of problem is a required field of study for computing the reliability of heat-supplying tunnels, yet there are no preceding studies on this matter.

Based on the operating Chegongzhuang tunnel, the improvement of the accuracy and efficiency of the reliability calculation of the tunnel lining structure has been made through the introduction of Hua-Wang point sets to subset simulation method. The mechanical response of the lining structure of a heat-supplying tunnel under the condition of multi-field coupling in the operation period was obtained for the first time. Ultimately, a new set of reliability analysis systems for operating heat-supplying tunnels has been established. The establishment of this system may provide some theoretical and technical supports for the hidden transformation of thermal underground engineering.

## 2. Testing Regime

### 2.1. General Situation of Heat-Supplying Engineering

**Figure 1:**
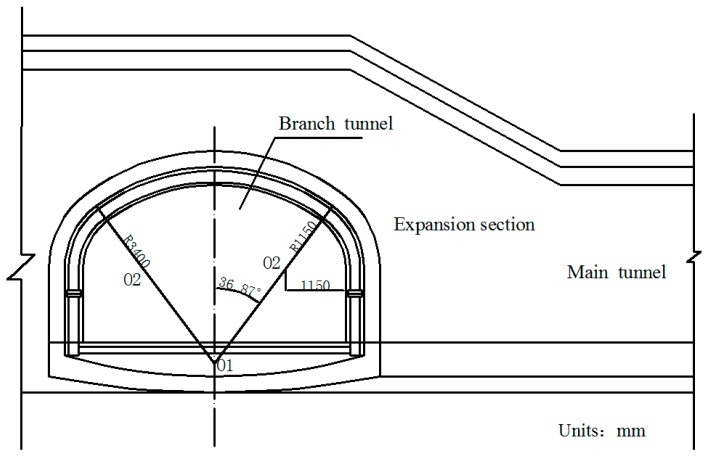
Sketch map of cross section of tunnels.

The operation tunnel of the west extension line of Chegongzhuang is located in Haidian District, Beijing. It starts from the Fuxing Road and ends at Fuchengmen Road, which passes through the Jingou River Road in the midway. The project was completed in August 2006 and has been in service for 11 years. The total length of the tunnel is about 1800 m. The buried depth of the floor of the composite lining reinforced concrete structure is 13.5 m. There is a heat-supplying tunnel branching off the main tunnel between Manholes 15 and 16. Both the main tunnel and the branch tunnel are built in the pebble boulder (Layer 3 in Table 1) based on the geological survey report from the design document. The properties of the three kinds of overlaying soil are listed in Table 1. The cross section is shown in Figure 1.

**Figure 2:**
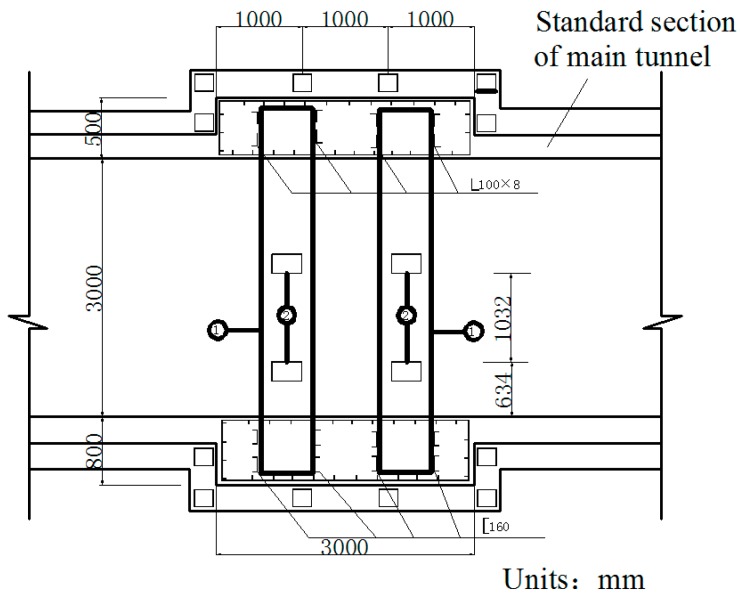
Thickened section on lining near the fixed brackets.

**Figure 3:**
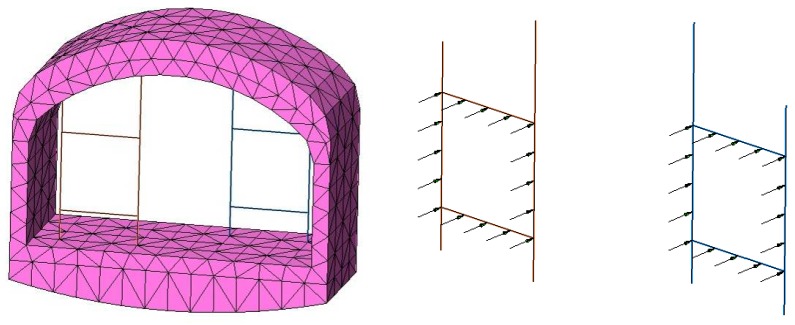
Fixed brackets and longitude thrust in a heat-supplying tunnel.

During operation, the fluid transfers heat to the pipe and its outer insulating layer through heat conduction. Because of the convection and radiation, the temperature of the inner wall of the tunnel rises and transfers heat to the outer wall and then to the surrounding rock. On the other hand, high-pressure fluid acts on the pressure in the pipe. Due to the limitation of the fixed bracket near the tunnel portal, the energy will act on the cross arm and the column of the fixed bracket in the form of a huge thrust and then the fixed bracket will transmit the force to the lining structure of the tunnel. Taking the Chegongzhuang tunnel as an example, the clearance size of the standard section of the main tunnel is 5.0 m (width) × 3.0 m (height). A set of fixed brackets in the tunnel is at the 1.5 m position of the north side of Manhole 15. The two fixed brackets are staggered 1 m front and back and the lining is thickened with 20 cm in 3 m range before and after the brackets’ zone as shown in Figure 2. The longitude thrust on the brackets is 2200 kN and 2150 kN respectively, pointing to the tunnel portal. The action diagram is shown in Figure 3.

### 2.2. Performance Functions and Variable Parameters

Based on the Chinese current design specification of concrete structure GB50010-2010 [18], the performance functions were set up. The general section of the lining of the tunnel was treated as the eccentric compression member. The large and small eccentricity was judged by Equation (1) at first and then Equations (2) and (3) were used respectively. In particular, in view of the longitudinal thrust response of Section 1, the longitudinal local bearing capacity of concrete Equation (4) was added.

(1) $$F_{1}=\frac{N-f_{y}^{\prime }A_{s}^{\prime }+f_{y}A_{s}}{bf_{\mathrm{cm}}}-\frac{0.8h_{0}}{1+\frac{f_{y}}{0.0033E_{s}}}$$

When F_1_ < 0, it belongs to the large eccentricity and uses Equation (2):

(2) $$F_{2}=(N-f_{y}^{\prime }As^{\prime }+f_{y}\mathrm{As})h_{0}-\frac{{\left(N-f_{y}^{\prime }As^{\prime }+f_{y}\mathrm{As}\right)}^{2}}{2bf_{\mathrm{cm}}}+f_{y}^{\prime }As^{\prime }(h_{0}-a_{s}^{\prime })-N(e_{0}+e_{a}+\frac{h}{2}-a_{s})$$

When F_1_ > 0, it belongs to the small eccentricity and uses Equation (3):

(3) $$\begin{array}{l} F_{3}=bf_{\mathrm{cm}}h_{0}\frac{N-f_{y}^{\prime }A_{s}^{\prime }+(0.0033E_{s}+f_{y})A_{s}}{bf_{\mathrm{cm}}+\frac{(0.0033E_{s}+f_{y})A_{s}}{0.8h_{0}}}-\frac{1}{2}bf_{\mathrm{cm}}{\left[\frac{N-f_{y}^{\prime }A_{s}^{\prime }+(0.0033E_{s}+f_{y})A_{s}}{bf_{\mathrm{cm}}+\frac{(0.0033E_{s}+f_{y})A_{s}}{0.8h_{0}}}\right]}^{2}+f_{y}^{\prime }A_{s}^{\prime }(h_{0}-a_{s}^{\prime })\\ -N(e_{0}+e_{a}+\frac{h}{2}-a_{s}) \end{array}$$

Particularly, the longitudinal local pressure performance function of the concrete in Section 1:

(4) $$F_{4}=f_{\mathrm{cm}}\cdot c\cdot 0.5h-N_{\mathrm{yy}}$$

where:

M_yy_, N_xx_ = the bending moment and axial force of lining structure during the operation period are derived from numerical simulation results;
N_yy_ = the longitudinal axial force of lining in Section 1, data from the said numerical simulation;
b = the width of lining cross section. b = 1 m;
c = the width of bracket cross section. c = 0.2 m;
e_0_ = the eccentricity of axial pressure on the center of gravity of the lining section, $e_{0}=\frac{M_{\mathrm{yy}}}{N_{\mathrm{xx}}}$; and
e_a_ = the additional eccentricity, 20 mm.

**Figure 4:**
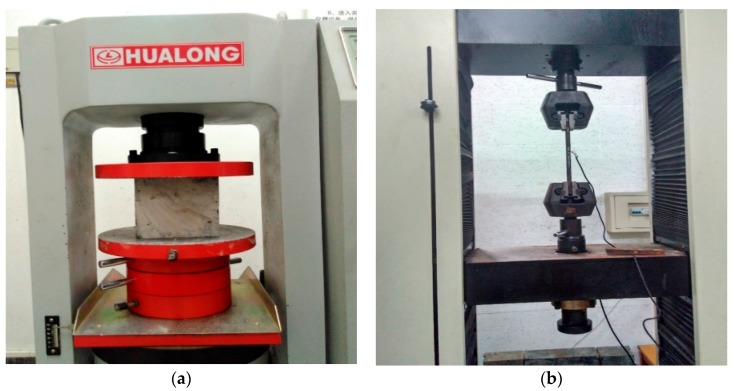
Mechanical property test of specimens. (**a**) Compressive strength test on concrete. (**b**) Tensile strength test on steel bars.

The variable parameters showed in Table 2 were all from the mechanical properties test of the in-situ specimens. Figure 4 showed compressive and tensile tests of concrete and steel bar specimens respectively. By testing the physical and mechanical properties of the concrete and steel bars of the lining structure, the real denaturation of the structural reliability can be determined.

### 2.3. Analysis Model and Basic Assumption

(1) The establishment of the analysis mechanism. In order to obtain the mechanical response of a heat-supplying tunnel under the coupling of temperature and large thrust during the operation period, the indirect analysis method was used in this paper. Firstly, the thermal analysis should be carried out to calculate the distribution of the temperature field. Secondly, the results of the thermal analysis are then applied to the model as the temperature load and the large thrust of the pipe. Finally, the internal force distribution of the lining structure in the operation period is obtained to provide internal force parameters for the performance function of reliability analysis.

Three-dimensional heat conduction partial differential equation [19]:

(5) $$\frac{\partial U}{\partial t}=\frac{k}{\rho c}(\frac{\partial U^{2}}{\partial^{2}x}+\frac{\partial U^{2}}{\partial^{2}y}+\frac{\partial U^{2}}{\partial^{2}z})$$

where:

U = temperature at any point;
t = time;
ρ = density;
c = specific heat capacity; and
k = thermal conductivity.

Xu Z.S. [17] gives the relationship between k, c and U:

(6) $$k\left(U\right)=1.6-\frac{0.6}{850}U$$

(7) $$c(U)=840+\frac{420U}{850}$$

Furthermore, this paper assumes that:

a. As far as the constitutive model was concerned, the concrete lining was a uniform linear elastic body and the soil was an elastoplastic body. The concrete lining and fixed bracket obeyed Hooke’s law and Table 3 offers the parameters of structure.

The soil mass obeyed the Mohr-Coulomb criterion. Mohr-Coulomb criterion can be expressed as [20,21]:

(8) $$\tau_{f}=c+\sigma \tan\varphi$$

where:

$\tau_{f}$ = the shear strength of soil;
σ = the vertical compressive stress on soil;
c = the cohesion of soil and φ = internal friction angle of soil, as shown in Table 1.

b. The gradual heating process in the early stage of heating was not considered. It was assumed that the inner wall of the lining was constant and the same. There was no heat loss between the outer wall of a tunnel and the soil. The temperature transition is uniform on the contact surface of the lining and the soil mass.
c. The saturation, porosity, soil moisture, thermal conductivity and the volume-specific heat of the soil never change with the change of the temperature in the calculation.
d. A finite soil model takes the place of an infinite soil model.

**Figure 5:**
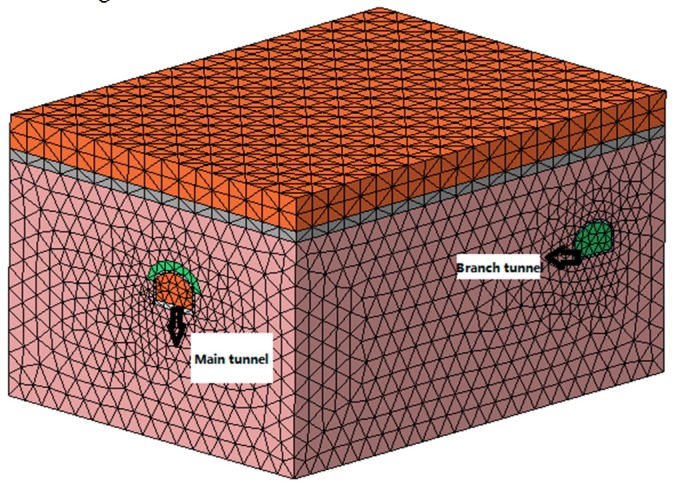
Numerical model grid.

(2) The establishment of the calculation model. The FLAC^3D^ three-dimensional finite difference program was used to simulate the tunnel in a certain range before and after the fixed brackets. The size of the numerical calculation model was 34 m(X) × 45 m(Y) × 24 m(Z). The fixed bracket was simulated by the beam element and the rest of the parts were simulated by four node tetrahedral entity elements, as shown in Figure 3 and Figure 5. 

**Figure 6:**
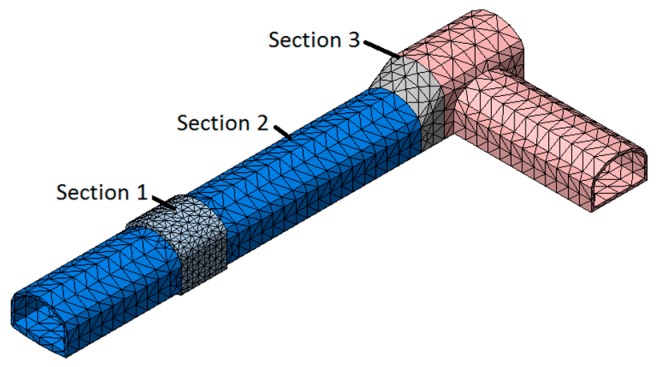
Tunnel lining grid and distribution of control sections.

According to the field monitoring, Figure 6 showed the distribution of control sections like the central section of the fixed brackets, Y = 0 m, Y = 12 m and the expansion section Y = 23 m. They were named Section 1, Section 2 and Section 3 by order.

(3) The selection of boundary conditions. 1. The mechanical boundary. Horizontal constraints were imposed on the vertical boundary. The edge sections of the tunnel were also limited by the horizontal constraints. Fixed constraints were imposed on the bottom boundary and the upper boundary was free. 2. The thermal boundary. It was measured that the temperature of the inner wall of the tunnel lining was 50 °C. According to the statistical research results in the literature [22], the temperature of land surface and initial boundary temperature of surrounding soil in the Beijing area were assumed 0 °C and 20 °C respectively. The distribution variation of the temperature value in the vertical direction and the influence of the heat-supplying operation of the branch tunnel were discarded. 

### 2.4. Field Monitoring 

**Figure 7:**
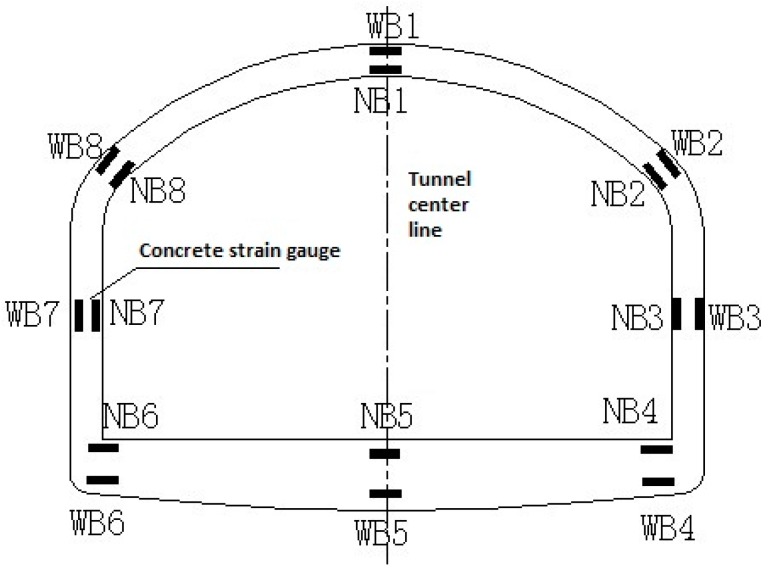
Layout of measuring points for each monitoring section.

Due to the need for excavation and dynamic monitoring, the team reserved survey points in the construction period of 2006. The longitudinal and lateral strain of the concrete were monitored by using concrete strain gauges, which were reserved for the lining within five sections of the Y = 0 m (Section 1), Y = 6 m, Y = 12 m (Section 2), Y = 18 m and Y = 23 m (Section 3). The layout of the monitoring points is shown in Figure 7.

## 3. Reliability Analysis System 

### 3.1. Subset Simulation Process

If G is the failure domain of performance function F(x): G = {x|F(x) ≤ 0}. By introducing the threshold value q_1_ > q_2_ > q_3_ > … > q_n_ = 0, a series of failure events G_k_ are formed. G_k_ needs to obey these rules:

(1) G_k_ = {x|F(x) ≤ q_k_} (k = 1,2,3,…,n);

(2) G_1_ ⊃ G_2_ ⊃ G_3_ ⊃…⊃ G_n_ = G;

(3) G_k_ = $\cap_{i=1}^{k}G_{i}$ (k = 1,2,3,…,n).

The failure probability can be expressed as a form of Markov chain as follows:

(9) $$P_{f}=P(G)=P(G_{1})\cdot \prod_{i=2}^{n}P(G_{i}|G_{i-1})$$

If P_1_ = P(G_1_), P_i_ = $P(G_{i}|G_{i-1})$, *P_f_* can also be expressed as Equation (10):

(10) $$P_{f}=\prod_{i=1}^{n}P_{i}$$

From Equation (10) we can know that when the order of magnitude of P_i_ is 10^−1^ and n = 5, the order of magnitude of P_f_ will be 10^−5^. As a result, the small probability problem can be transformed into the product of the larger conditional probability by using the subset simulation method, which can improve the efficiency of estimation of a small failure probability event.

#### 3.1.1. Select the Intermediate Failure Event

From the theory of the subset simulation method, failure event can be expressed as a set of a series of intermediate failure events like G_1_,G_2_,G_3_,…,G_n_. Therefore, it was very important to select appropriate intermediate failure events when using the subset simulation method to calculate the reliability problems. Au put forward a preset value of conditional probability P_0_ and the method of automatic segmentation, in which the number of sampling points of each layer need to be the same [1,2]. Like N_i_ = N_0_, both P_0_N_0_ and (1–P_0_)N_0_ must be integers.

#### 3.1.2. Algorithm Implementation

**Figure 8:**
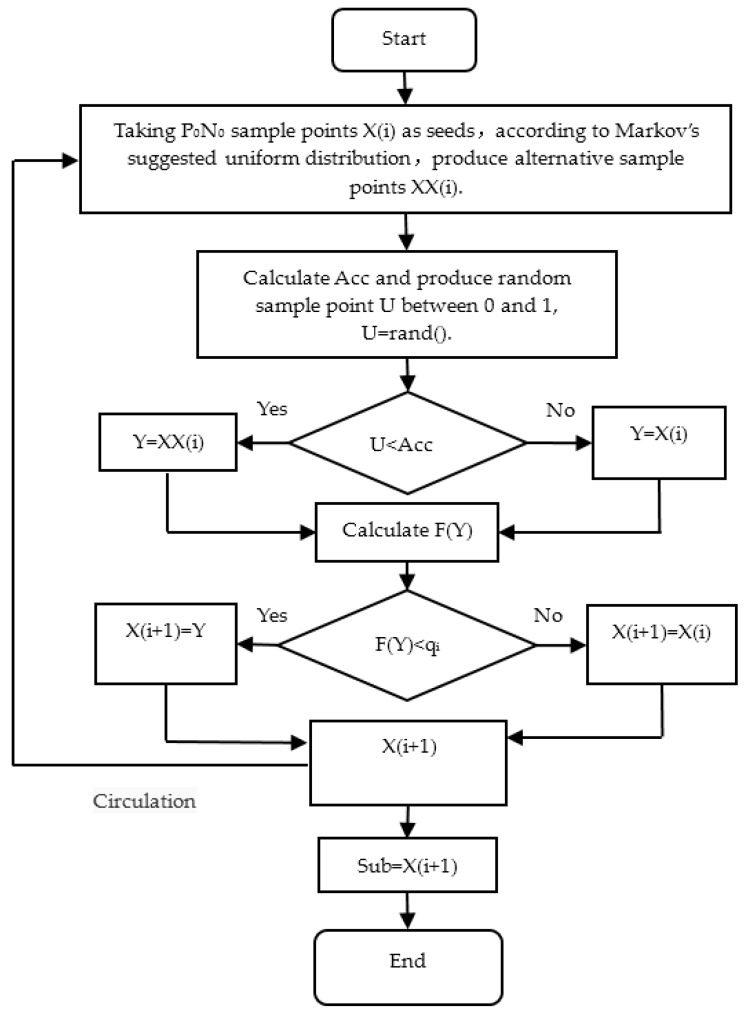
Subroutine flow chart of Markov chain in subset simulation.

Based on the principle of subset simulation method, the automatic hierarchical process of subset simulation was shown as follows:

(1) The Carlo Monte method was used to generate *N* sample points $\left\{X_{j}^{\left(1\right)}:j=1,2,3,\ldots ,N_{0}\right\}$. These sample points were independent and obeyed the basic random variables of the probability density function $f_{x}(X)$.
(2) Calculate the function values F($x_{j}^{\left(i\right)}$) $\left(j=1,2,3,\ldots ,N_{0}\right)$ corresponding to the number of N_0_ sample points respectively. In descending order by the function of value, take the number (1–P_0_)N_0_ value as the critical value q_1_ of failure event G_1_. Here, q_1_ = F($x_{\left(1-p_{0}\right)N_{0}}^{\left(1\right)}$) and P_1_ = P_0_.
(3) Starting from the number of P_0_N_0_ sample points that fell within the $G_{i-1}(i=2,3,4,\ldots ,n)$, each sample point can be simulated by a Markov chain. Therefore, the number of $1/P_{0}$ sample points were generated by each Markov chain. Therefore, the number of samples generated per layer was maintained at N_0_. The specific process was shown in Figure 8, where transfer acceptance rate of Markov chain Acc = min{1,$\frac{F(XX(i))}{F(X(i))}$}.
(4) Analog the second step and use the N sample points captured by the third step to calculate the value F($x_{j}^{\left(i\right)}$) of each function. In descending order, take the number (1–P_0_)N_0_ value as the critical values q_i_ of failure event G_i_. Here, q_i_ = F($x_{\left(1-p_{0}\right)N_{0}}^{\left(i\right)}$) and P_i_ = P_0_.
(5) Repeat Step (3) and Step (4), until the critical value q_n_ in Layer l was less than 0. Ultimately, the number of failure events was N_f_ in N_0_ sample points. Therefore, the conditional probability estimated: $P_{n}=\frac{N_{f}}{N_{0}}$.
(6) Final failure probability estimated: ${\hat{P}}_{f}=P_{0}{}^{n-1}P_{n}=P_{0}{}^{n-1}\cdot \frac{N_{f}}{N_{0}}$.

### 3.2. Problems of the General Subset Simulation Method and Its Improved Method

#### 3.2.1. Problems of the Existing Method

The N_0_ sample points are generated by the Monte Carlo method in the general subset simulation method. These sample points are pseudorandom numbers, which has a certain correlation. The distribution of the sample points is not uniform and the deviation can reach $ο(n^{-0.5})$ [23]. For the calculation of small probability events represented by the structural failure probability, the convergence speed and accuracy are affected by PRN sampling points. On the one hand, the low convergence speed determines a huge time consuming in calculation. Especially in the reliability analysis of an underground structure engineering, the operation time cost may reach ten hours or even days without the subset simulation process. On the other hand, the reliability of the calculation results can be doubtful because of the accuracy problems.

#### 3.2.2. Improved Subset Simulation Method

In number theory, the Hua-Wang point set is a low deviation point set, which has good uniformity [23]. $\gamma =(\gamma_{1},\gamma_{2},\ldots ,\gamma_{m})\in R^{m}$, if P = {$\left(\gamma_{1}k),(\gamma_{2}k),....,(\gamma_{m}k\right)$} (k = 1,2…) and the former n items have a deviation $D(n,P_{n})\leq c(\gamma ,\xi )\cdot n^{-1+\xi }$, then P_n_ can be called a good point set and γ is a good point.

(11) $$\gamma =[\{2\cos\frac{2\pi }{p}\},\{2\cos\frac{4\pi }{p}\},....,\{2\cos\frac{2\pi m}{p}\}]$$

where p should be a prime number and p≥2m+3. The point set P_n_ has a deviation $ο(n^{-0.5-\frac{1}{2(m-1)}+\xi })$.

**Figure 9:**
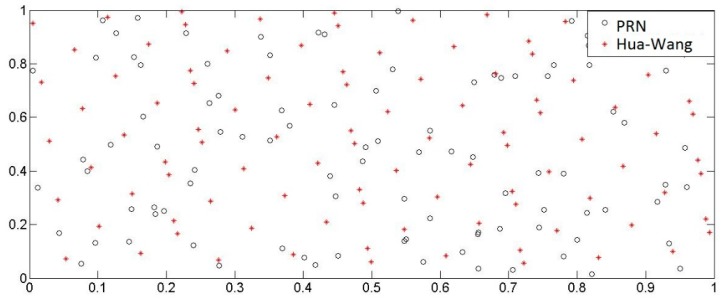
Distribution of 100 sample points generated by the two methods.

One hundred sample points generated randomly in two-dimensional unit space by using the Hua-Wang point set and the PRN method respectively were shown in Figure 9. It is found that the point distribution generated by the Hua-Wang point set method is more uniform. Therefore, the improvement of the subset simulation algorithm is embodied in Step (1) of Section 3.1.2 by using the Hua-Wang point set instead of Monte Carlo’s PRN to generate more independent and uniformly distributed sample points.

## 4. Results and Discussion

### 4.1. Numerical Simulation 

**Figure 10:**
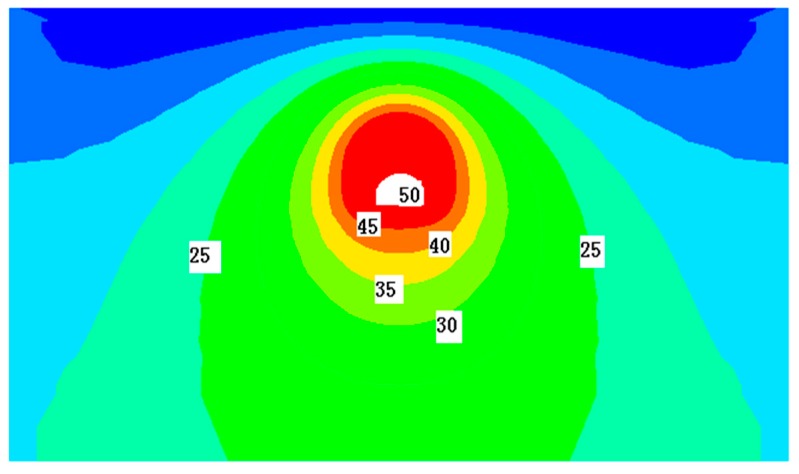
Temperature field distribution of the main tunnel (Units: °C).

(1) Before the operation period, according to the construction steps, the excavation and support of the main tunnel, the expansion section and then the branch tunnel were simulated. After the installation of the fixed brackets, the state of the main tunnel before heating was obtained. Because of the thickening of the lining, the maximum principal stress $\sigma_{1}$ of Section 1 is smaller in Table 4. The longitudinal strain of Section 1 and Section 2 is very small, which is in line with the plane strain characteristics of tunnel mechanics. The maximum tensile stress of the spandrel of Section 3 is up to 2.1 MPa, which is greatest affected by the excavation of the expansion section and branch tunnel among three sections.

(2) During the operation period, based on the indirect analysis method in said material, first consider the effect of temperature field separately. We can get the temperature field distribution by numerical calculation. The temperature field distribution of the main tunnel standard section (Section 2) is shown in Figure 10. In Figure 10, the crown and spandrel of the tunnel structure are dense areas of equal value, which indicates that the temperature gradient in crown and spandrel is large. In other words, the temperature difference between inner and outer lining at crown and spandrel is high. Because the shallow tunnel is greatly influenced by the land surface temperature, the temperature difference can reach 4.9 °C and spandrel is 4.6 °C. Against this backdrop, the temperature difference between the inner and outer walls is the cause of the temperature stress in the tunnel structure and the development of the relevant temperature crack should be prevented.

(3) Then, after applying thermal load and large thrust of pipeline, the mechanical response of lining structure of heat-supplying tunnel under the coupling condition of large thrust and temperature field during operation is obtained. The difference between before and after heating in Table 4 can be seen as the characterization of the coupling effect. Table 4 shows that because the huge pipe thrust is directly on the fixed bracket, it has a great influence on Section 1. The minimum principal stress of the floor in Section 1 is increased from −14.3 MPa to −17.8 MPa. The longitudinal effect is particularly prominent and the longitudinal compressive strain ε_yy_ reaches the −56.7 με. Furthermore, the stress factor applies the greatest influence on the floor, the crown and on the side wall in the respective order. Comparing the results before and after operation, the variation of longitudinal stress and strain of Section 2 is less and within 5.5 με at each place. The lateral strain changes in the range of 3 με. It shows that the coupling effect has been markedly attenuated at the distance 12 m from the bracket. For Section 3, there is little change in the longitudinal strain before and after operation. It can be seen that for the lining structure away from the fixed bracket, the influence of the coupling effect is mainly reflected by the single temperature field in the form of circumferential stress and strain. Results show that its influence is very limited and its maximum lateral strain can reach −5.6 με at the foot of sidewall. According to the results of numerical simulation, the internal force distribution of the lining structure can be obtained as Table 5 shows. It can be plugged into the performance functions in reliability calculation. The results indicate that due to the multi-field coupling effect in the operation period, the design of the heat-supplying tunnel at the present stage is blind only by enlarging the whole section thickness. The floor of the tunnel under the fixed bracket should be the key part. 

### 4.2. Field Monitoring

**Figure 11:**
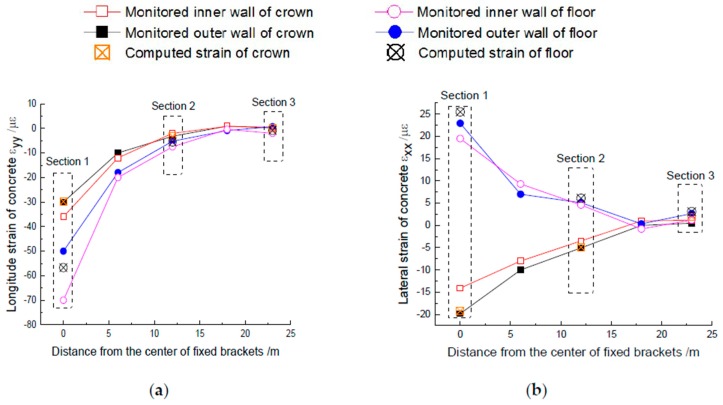
Monitoring results of strain variation of concrete under coupling. (**a**) Longitudinal strain variation of concrete; (**b**) Lateral strain variation of concrete.

Corresponding to the changes in the longitudinal and lateral strain of concrete before and after the operation respectively, Figure 11 shows the measured value of the coupling effect on the concrete strain. It can be found that: 1. the variation of longitudinal and lateral strain on concrete is roughly consistent with the increase of distance from the center of the fixed brackets and the decrease of strain. In the range of 6 m, the longitudinal strain on the floor attenuates by about 60% and the lateral strain attenuates by 50%. Therefore, the 6 m range of the fixed bracket can be considered as the focus of monitoring and protection in the operation period of the heat-supplying tunnel and the coupling effects can be less beyond 6 m. 2. For longitudinal strain, taking the floor as an example, the average strain of the measured floor in Section 1, Section 2 and Section 3 is −65 με, −6.4 με and −0.7 με respectively and the results of numerical simulation are −56.7 με, −5.5 με and −0.8 με. 3. Similarly, for lateral strain, the average strain on the floor is 21.2 με, 4.9 με and 1.9 με respectively and the results of numerical simulation are 25.5 με, 6.1 με and 3.1 με. 4. Comparing the computed results with the monitored results, it is not difficult to find that the results of the numerical simulation are consistent with the measured laws in general. Therefore, the reasonableness of the numerical simulation in said materials has been verified and its result can be used for reliability calculation and analysis.

### 4.3. Failure Probability and Reliability Index 

In the study of reliability, it is generally believed that when the number of samples is large enough, the accuracy of the Monte Carlo method can be high enough to be a contrast standard. When the general subset simulation method and its improved method are used, the conditional failure probability P_0_ = 0.1 and 1000 sample points were pumped into each layer. The failure probability of each section and the corresponding reliability index beta were obtained. The results were shown in Table 6, compared with the traditional Monte Carlo method.

The results of Section 1 and Section 2 in Table 6 show that the floor near the fixed bracket of the heat-supplying tunnel is the section with the lowest reliability in the structure. The reliability index of the floor in Section 1 calculated by Monte Carlo method, improved subset simulation method and general subset simulation method are 3.04, 2.93 and 2.91 respectively. For the general heat-supplying underground structure, the target design reliability index β_t_ = 3.2. In engineering, 0.85β_t_ = 2.72 is generally used as a control reliability index of the tunnel [24]. Therefore, it is not difficult to find the great influence of the coupling effect on the reliability of the floor of the heat-supplying tunnel and it is necessary to reinforce the floor near the fixed bracket.

### 4.4. Evaluation of Reliability Analysis Methods

#### 4.4.1. Accuracy and Dispersion

**Figure 12:**
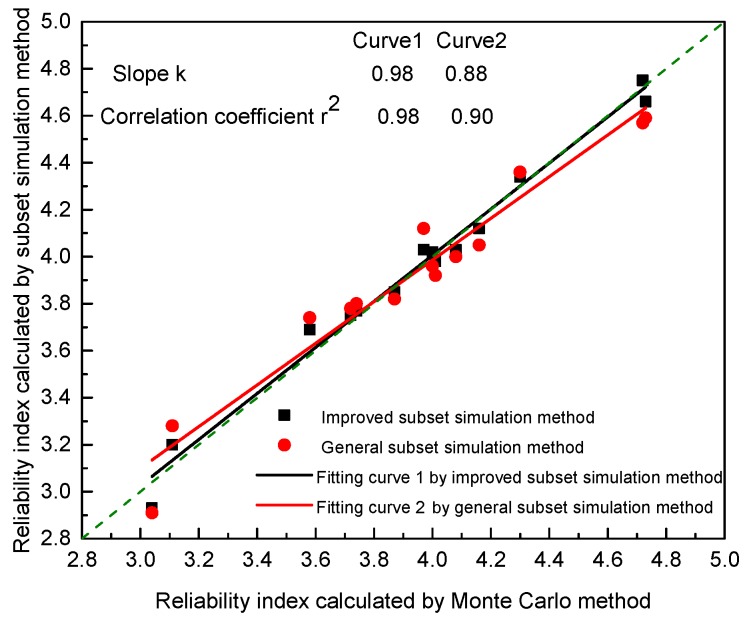
Contrast analysis of the calculation errors of the methods.

Accuracy and dispersion are the most important evaluating indicators of a reliability analysis method. The reliability indexes of all the sections calculated in Table 6 are extracted to integrate and contrast the calculation errors of the general subset simulation method and its improved method, as shown in Figure 12. 

In Figure 12, the scatter points of the improved subset simulation approach are denser, approaching the diagonal line with a slope of 1. The slope of the fitting curve k reaches 0.98 over the general method’s 0.88 and the correlation coefficient 0.98 is also greater than 0.9. It shows that the accuracy of the improved method is higher and the degree of dispersion is lower. 

The key to the success of the improved method lies in the low deviation of the Hua-Wang point set. The existing methods, such as Monte Carlo and general subset simulation, are based on PRN. Table 6 indicates that the number of sample points required for the general subset simulation method and the improved method is in the range of 3000 to 6000 in this study. A fewer number of samples were derived from the reliability analysis. The six random variable parameters in Table 2 brings a high dimension problem for PRN. High dimension and small sample size can lead to the distribution of PRN points inconsistent with the true distribution. Against this backdrop, the general subset simulation method has a higher probability of producing erroneous results than that of the improved method as Table 6 shows. 

These cases indicate that the subset simulation method improved by the introduction of the Hua-Wang point set is optimized in the calculation error. It could be a preferred method to ensure accuracy and dispersion.

#### 4.4.2. Economic Efficiency

**Figure 13:**
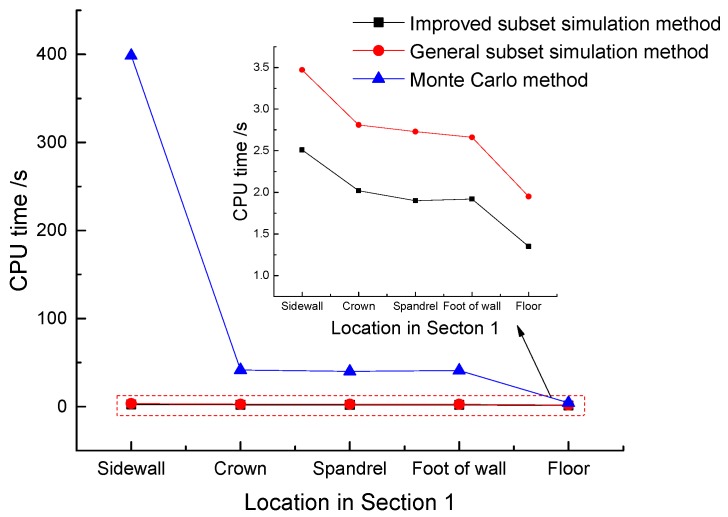
Contrast analysis of the calculation efficiency of the methods.

Economic efficiency is also an important evaluating indicator for the improvement of any reliability calculation methods. Reducing the sample quantity and improving the sampling efficiency can be the basis for increasing economic efficiency. The CPU time calculated by each method of Section 1 is extracted as Figure 13.

(1) Sampling quantity. The scientific counting method is generally used to record the failure probability P_f_, P_f_ = a × 10^−b^. The number of required sample points is usually between 10^b+1^ ~ 10^b+2^ in accordance with the experience of the existing Monte Carlo sampling times, N_M−C_ ≥ 10^b+1^. Looking at the algorithm principle and Table 6, it can be concluded that the subset simulation method and its improved method need a sample number of N_SS_ = N_0_ × b. Therefore, the analytic relation between the number of sample points N_SS_ and the number of Monte Carlo sample points N_M-C_ for the subset simulation method is as follows:

N_SS_ ≤ N_0_·(lgN_M-C_ − 1) (12)

where, N_0_ is the number of sampling points per layer in the subset simulation method; N_0_ = 1000 in this paper.

In Table 6, the failure probability of the sidewall is the lowest and that of the floor is the highest. In Figure 13, the maximum time utilization ratio (TUR) of the improved subset simulation method at the side wall can reach 158.8. The number of sampling points in the Monte Carlo method increases sharply when the failure probability is low, while the sample points needed by the subset simulation method increase little. Under these circumstances, the efficiency of the improved method is significantly reflected. However, owing to the required number of sample points by these three methods at the floor of tunnel is not large, the advantage of subset simulation is not obvious because of the complexity of per step logic operation.

(2) Sampling efficiency. From a global point of view, general subset simulation and the improved subset simulation have the same number of sampling points. Therefore, the efficiency of the sampling method determines the whole calculation efficiency. The results obtained in Figure 13 actually show the difference in efficiency of Hua-Wang point set and PRN sampling method. The analysis results suggest that in a six-dimensional space of 1000 sample points per layer, PRN method has a greater disadvantage in convergence speed than Hua-Wang point set. The improved method can save computing time to the reduction of 27.7%, 28.1%, 30.4%, 27.8% and 30.8% respectively at the locations in Section 1. In the analysis of the reliability of complex tunnel structures, the benefits “Lower deviation and correlation in small scale sample points high dimensional space” of Hua-Wang point set contribute to the economic efficiency more prominently. 

In a complex underground engineering, a great many sections demand to be selected and the performance functions are repeatedly invoked. It often takes more than ten hours or even days to operate on a microcomputer when using the PRN sampling method. The above mentioned results prove that the subset simulation method modified by the Hua-Wang point set can be a preferred method to ensure the economic efficiency.

## 5. Conclusions

In this paper, preliminary conclusions and recommendations are as follows:

(1) The multi-field coupling effects in the operation period attenuate with the increase of the distance between the structure section and the fixed bracket. The tunnel lining structure in the range of the fixed bracket at 6 m is greatly influenced by the coupling and the floor has the lowest reliability. In the process of design, construction and maintenance of heat-supplying underground structures, special design and protective measures for the structural floor of the fixed bracket 6 m should be put forward according to the operation characteristics of heat-supplying engineering.

(2) This paper improves subset simulation based on Hua-Wang point set. The results show that this method can save computation time and improve computing efficiency on the premise of guaranteeing the computation error and its efficiency is more significant when the failure probability is lower. Considering that the failure probability of tunnel engineering is generally small, the improved subset simulation method has a certain application prospect in the calculation of tunnel engineering reliability.

(3) The reliability analysis system of the heat-supplying tunnel in the operation period established in this paper can provide some theoretical support for the hidden transformation of the general heat-supplying underground engineering.

## Figures and Tables

**Table 1:** Soil layer information.

Item	Name	Density (kg/m^3^)	Specific Heat Capacity (J/(kg·K))	Thermal Conductivity (W/(m·K))	Modulus of Elasticity (GPa)	Poisson Ratio	Thickness (m)	Cohesion (kPa)	Internal Friction Angle (°)	Data Sources
Layer 1	Miscellaneous fill	1920	1010	1.44	0.0086	0.30	2	5	22	Geological survey report
Layer 2	Sand	1970	930	1.51	0.03	0.28	1	0	30	
Layer 3	Pebble boulder	2070	960	1.61	0.04	0.30	21	0	30	

**Table 2:** Distribution of variable parameters.

Item	Symbol (Unit)	Mean Value	Standard Error	Distribution Form	Data Sources
Apparent tensile strength of main steels	f_y_ (MPa)	270	10.1	Normal distribution	Indoor test
Diameter of main steels	d (mm)	19.1	0.9	Normal distribution	Sampling measurement
Distance between the resultant force point of the main reinforcement and the compression edge	a_s_ (mm)	27	5	Normal distribution	Nondestructive test
Distance between the point of action of the main force of the main bar of the drawing area to the drawing edge	a_s_ (mm)	25	6	Normal distribution	Nondestructive test
Concrete strength	f_cm_ (MPa)	27.2	8.1	Normal distribution	Indoor test
Modulus of elasticity of main steels	E_s_ (GPa)	180	12	Lognormal distribution	Indoor test

**Table 3:** Parameters of structure.

Item	Material	Density (kg/m^3^)	Specific Heat Capacity (J/(kg·K))	Thermal Conductivity (W/(m·K))	Modulus of Elasticity (GPa)	Poisson Ratio	Data Sources
Lining	Reinforced concrete	2400	Equation (7)	Equation (6)	30	0.25	Design acceptance document
Fixed bracket	Steel	7850	460	14.7	200	0.2	

**Table 4:** Stress and strain situation of each control section before and after operation.

Location		Section 1				Section 2				Section 3			
		Principal Stress/MPa		Strain/με		Principal Stress/MPa		Strain/με		Principal Stress/MPa		Strain/με	
		$\sigma_{1}$	$\sigma_{3}$	$\varepsilon_{xx}$	$\varepsilon_{yy}$	$\sigma_{1}$	$\sigma_{3}$	$\varepsilon_{xx}$	$\varepsilon_{yy}$	$\sigma_{1}$	$\sigma_{3}$	$\varepsilon_{xx}$	$\varepsilon_{yy}$
Crown	Before operation	−0.2	−2.1	−40.1	2.9	−1.7	−5.4	−77.2	2	−1.9	−9	−100.1	−5.5
	After operation	1.1	−4.3	−59.3	−26.9	−1.5	−6.1	−82.1	−1.3	−1.85	−8.9	−98.2	−5.3
	Difference	1.3	−2.2	−19.2	−29.8	0.2	−0.7	−4.9	−3.3	0.05	0.1	1.9	0.2
Spandrel	Before operation	0.5	−5.2	−80.1	−0.9	0.03	−5.4	−81.6	−0.8	2.1	−4.2	60.3	4.1
	After operation	−0.5	−6.1	−70.9	−13.1	−0.1	−4.5	−77.9	−3.6	2.12	−3.6	65.5	4.4
	Difference	−1	−0.9	9.2	−12.2	−0.13	0.9	3.7	−2.8	0.02	0.6	5.2	0.3
Sidewall	Before operation	−0.3	−2.1	−12.6	1.1	−0.5	−1.4	−12.6	−1.1	−0.1	−3.3	−35.0	−3.6
	After operation	−0.8	−4.1	−30.1	−10.7	−0.7	−2	−17.1	−4.4	−0.08	−2.9	−33.6	−3.1
	Difference	−0.5	−2	−17.5	−11.8	−0.2	−0.6	−4.5	−3.3	0.02	0.4	1.4	0.5
Foot of wall	Before operation	0.4	−3.3	6.9	2.3	0.6	−2.3	8.9	−2.5	0.6	−3.9	13.2	−5.1
	After operation	1	−3.1	17.1	−14.5	0.7	−3.4	10.1	2	0.5	−3.2	7.6	−5.6
	Difference	0.6	0.2	10.2	−16.8	0.1	−1.1	1.2	4.5	−0.1	0.7	−5.6	−0.5
Floor	Before operation	0.6	−14.3	−440.1	−1.5	1	−14	−381.6	−1.5	1.1	−4.2	33.6	2.4
	After operation	2.6	−17.8	−414.6	−58.2	1.2	−12.8	−375.5	−7	1.2	−4.1	36.7	1.6
	Difference	2	−3.5	25.5	−56.7	0.2	1.2	6.1	−5.5	0.1	0.1	3.1	−0.8

In this paper, the tensile stress and strain are positive and the compressive stress and strain are negative.

**Table 5:** Internal force distribution of lining structure.

Section	N_xx_ (kN)	M_yy_ (kN·m)
Section 1	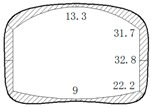	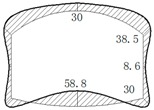
Section 2	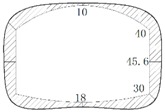	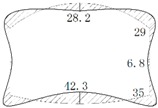
Section 3	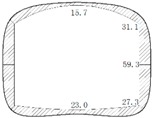	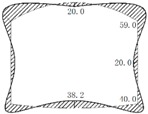

**Table 6:** Comparison of the failure probability among the traditional Monte Carlo, the subset simulation and its improved method.

Location and Section	Improved Subset Simulation Method (General Subset Simulation Method)				Monte Carlo Method				Error of Reliability Index $|\beta_{1}-\beta_{0}|/\beta_{0}$/%	Time Utilization Ratio (TUR) T_0_/T_1_
	Failure Probability P_f_	Number of Required Sample Points N_SS_	CPU Time T_1_/s	Reliability Index *β*_1_	Failure Probability P_f_	Number of Sample Points N_M-C_	CPU Time T_0_/s	Reliability Index β_0_		
Crown in Section 1	2.8 × 10^−5^ (1.9 × 10^−5^)	5000	2.02 (2.81)	4.03 (4.12)	3.6 × 10^−5^	1,000,000	41.58	3.97	1.5 (3.8)	20.6 (14.8)
Spandrel in Section 1	8.3 × 10^−5^ (7.3 × 10^−5^)	5000	1.90 (2.73)	3.77 (3.80)	9.3 × 10^−5^	1,000,000	40.05	3.74	0.8 (1.6)	21.1 (14.7)
Sidewall in Section 1	1.0 × 10^−6^ (2.4 × 10^−6^)	6000	2.51 (3.47)	4.75 (4.57)	1.2 × 10^−6^	10,000,000	398.48	4.72	0.6 (3.2)	158.8 (114.8)
Foot of wall in Section 1	3.5 × 10^−5^ (4.4 × 10^−5^)	5000	1.92 (2.66)	3.98 (3.92)	3.0 × 10^−5^	1,000,000	40.93	4.01	0.7 (2.2)	21.3 (15.4)
Floor in Section 1	1.7 × 10^−3^ (1.8 × 10^−3^)	3000	1.35 (1.95)	2.93 (2.91)	1.2 × 10^−3^	100,000	4.35	3.04	3.6 (4.3)	3.2 (2.2)
Crown in Section 2	2.8 × 10^−5^ (3.1 × 10^−5^)	5000	1.99 (2.83)	4.03 (4.00)	2.3 × 10^−5^	1,000,000	40.26	4.08	1.2 (2.0)	20.2 (14.2)
Spandrel in Section 2	2.9 × 10^−5^ (3.7 × 10^−5^)	5000	1.91 (2.78)	4.02 (3.96)	3.2 × 10^−5^	1,000,000	42.03	4.00	0.5 (1.0)	22.0 (15.1)
Sidewall in Section 2	1.6 × 10^−6^ (2.2 × 10^−6^)	6000	2.71 (3.79)	4.66 (4.59)	1.1 × 10^−6^	10,000,000	396.46	4.73	1.5 (3.0)	146.3 (104.6)
Foot of wall in Section 2	5.9 × 10^−5^ (6.6 × 10^−5^)	5000	2.03 (2.90)	3.85 (3.82)	5.5 × 10^−5^	1,000,000	42.36	3.87	0.5 (1.3)	20.9 (14.6)
Floor in Section 2	1.1 × 10^−4^ (9.1 × 10^−5^)	4000	1.62 (2.41)	3.69 (3.74)	1.7 × 10^−4^	1,000,000	42.13	3.58	3.1 (4.5)	26.0 (17.5)
Crown in Section 3	1.9 × 10^−5^ (2.6 × 10^−5^)	5000	1.99 (2.92)	4.12 (4.05)	1.6 × 10^−5^	1,000,000	39.56	4.16	1.0 (2.6)	19.9 (13.5)
Spandrel in Section 3	6.9 × 10^−4^ (5.2 × 10^−4^)	4000	1.69 (2.45)	3.20 (3.28)	9.2 × 10^−4^	1,000,000	39.40	3.11	2.9 (5.5)	23.3 (16.1)
Sidewall in Section 3	7.0 × 10^−6^ (6.4 × 10^−6^)	6000	2.70 (3.59)	4.34 (4.36)	8.7 × 10^−6^	10,000,000	360.14	4.30	0.9 (1.4)	133.4 (100.3)
Foot of wall in Section 3	8.8 × 10^−5^ (7.9 × 10^−5^)	5000	2.05 (2.93)	3.75 (3.78)	9.8 × 10^−5^	1,000,000	35.84	3.72	0.8 (1.6)	17.5 (12.2)
Floor in Section 3	8.3 × 10^−5^ (7.1 × 10^−5^)	5000	1.97 (2.88)	3.77 (3.80)	9.2 × 10^−5^	1,000,000	36.85	3.74	0.8 (1.6)	18.7 (12.8)

The computer configuration used for computing: CPU, Intel Core i7-6700@3.40Hz, RAM: 16.0 GB. The comparison calculation is based on the Monte Carlo method as the reference standard.
